# Case Report: Recurrent metastatic thymoma-associated multiple paraneoplastic syndromes: myasthenia gravis, dysgeusia, and acute intestinal pseudo-obstruction

**DOI:** 10.3389/fonc.2026.1937358

**Published:** 2026-09-04

**Authors:** Yunlong Guo, Guangxin Zhang, Chengyan Jin

**Affiliations:** Department of Thoracic Surgery, Second Hospital of Jilin University, Changchun, China

**Keywords:** acetylcholine receptor antibody, acute intestinal pseudo-obstruction, CAP chemotherapy regimen, dysgeusia, myasthenia gravis, paraneoplastic syndrome, thymoma recurrence

## Abstract

**Background:**

Paraneoplastic syndromes (PNS) are frequently associated with thymoma. The unusual clustering of myasthenia gravis, dysgeusia, and AIPO during thymoma recurrence has been sparsely reported in the literature.

**Case presentation:**

We report the case of a man who developed pleural metastasis three years after thymoma resection and shortly thereafter experienced dysgeusia, ocular MG (diplopia and ophthalmoplegia), and AIPO. This triad has received limited documentation. Serum testing was positive for acetylcholine receptor antibody (AChR-Ab), anti-MURC antibody, and anti-recoverin antibody. After combined CAP chemotherapy and symptomatic pyridostigmine therapy, the pleural lesions regressed, and the ocular, taste, and gastrointestinal symptoms also resolved.

**Conclusion:**

This case illustrates that multisystem PNS can arise years after thymectomy and may cluster, emphasizing the need for early recognition and multidisciplinary, comprehensive management.

## Introduction

Approximately half of patients with thymoma develop paraneoplastic syndromes (PNS) (1), and about one-fifth of cases either present for the first time or recur in association with tumor relapse years after thymectomy (2). Myasthenia gravis (MG) is the most common manifestation, accounting for 30% to 44% of thymoma-associated PNS (3). However, only 4% to 7% of patients with thymoma-associated MG develop additional PNS, such as autoimmune autonomic neuropathy, neuromyotonia, limbic encephalitis, or other organ-specific autoimmune diseases (4). In the published literature, thymoma-associated MG accompanied by acute intestinal pseudo-obstruction (AIPO) or dysgeusia has received limited documentation, with 12 reported cases of MG with AIPO (5) and approximately 15 to 20 reported cases of MG with dysgeusia (6–8). Since the 1990s, only two cases describing the full triad of MG, AIPO, and dysgeusia have been reported worldwide (9). Here, we report a case of concurrent triple PNS associated with pleural metastatic thymoma. This case is presented to increase awareness that thymoma-related PNS can be delayed in onset, multisystem in scope, clustered in presentation during recurrence and demonstrated regularity in recovery, and to highlight the importance of early recognition and coordinated multidisciplinary care.

## Case presentation

**Figure 1:**
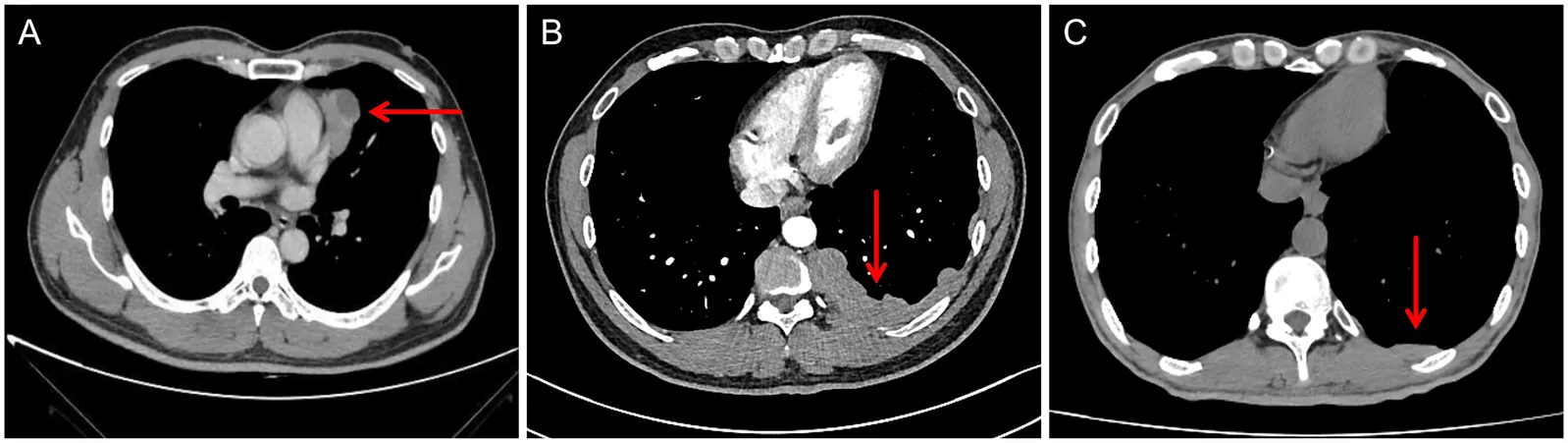
**(A)** (indicated by the arrow) shows a mass-like, irregularly-margined hypodense lesion measuring approximately 51 mm×35 mm in the anterior mediastinum adjacent to the pericardium, with an internal roundish low-density area, as revealed by preoperative axial contrast-enhanced chest CT. **(B)** (indicated by the arrow) shows significant thickening of the left pleura on pre-chemotherapy axial contrast-enhanced chest CT, which was considered metastatic. **(C)** (indicated by the arrow) shows a follow-up axial chest CT obtained after the completion of all chemotherapy cycles, demonstrating a marked reduction in both the degree of left pleural thickening and the extent of the lesion compared to the pre-chemotherapy findings.

**Figure 2:**
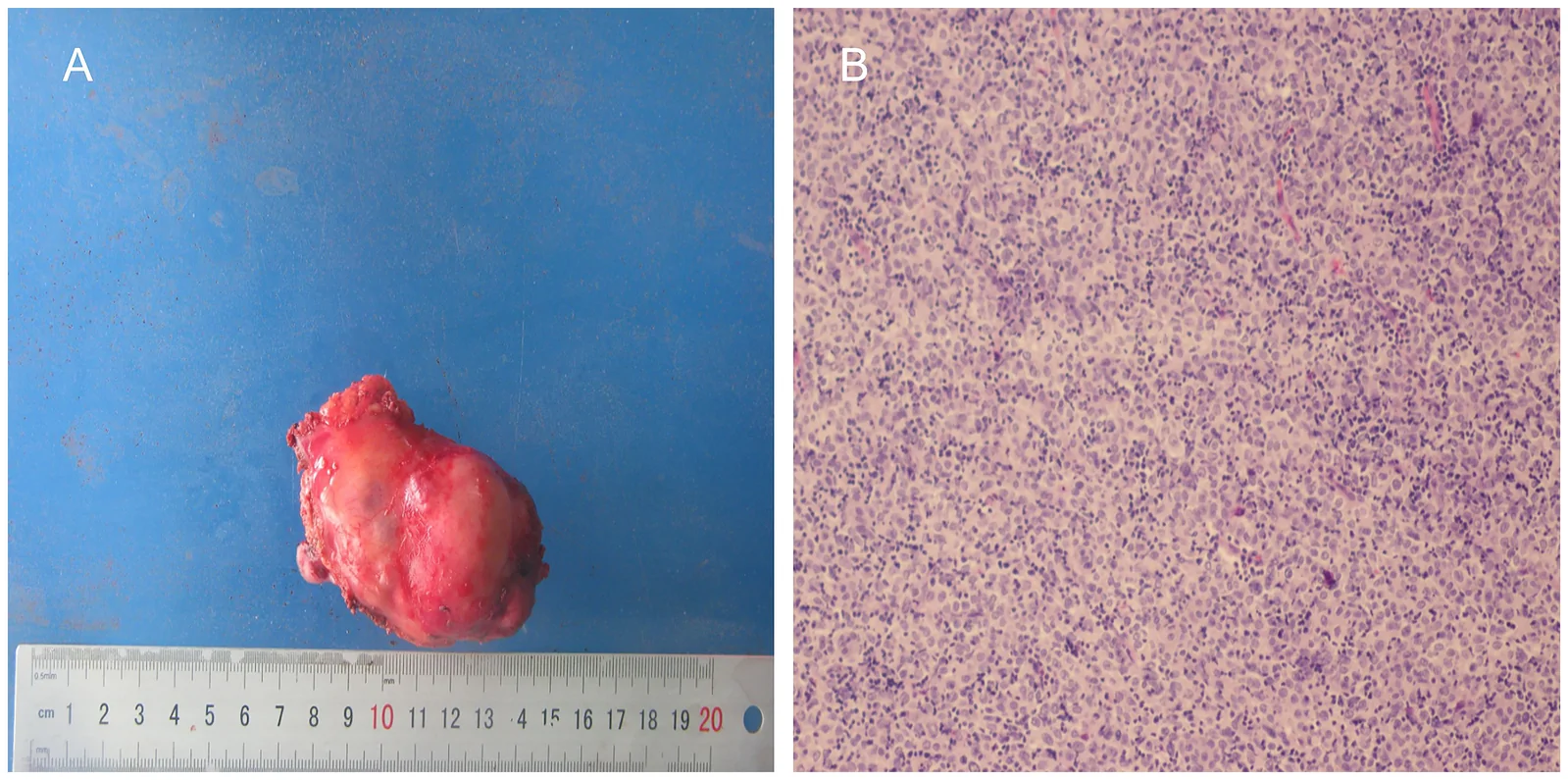
**(A)** Shows the gross specimen of the resected thymic tumor. **(B)** Shows the microscopic aspect of thymic tumor, with neoplastic epithelial cells arranged in nests and sheets, characterized by large vesicular nuclei and prominent nucleoli, against a background of lymphoid stroma infiltrated by numerous reactive lymphocytes with small dense nuclei. ×200 enlargement, hematoxylin and eosin (H&E) stain.

**Figure 3:**
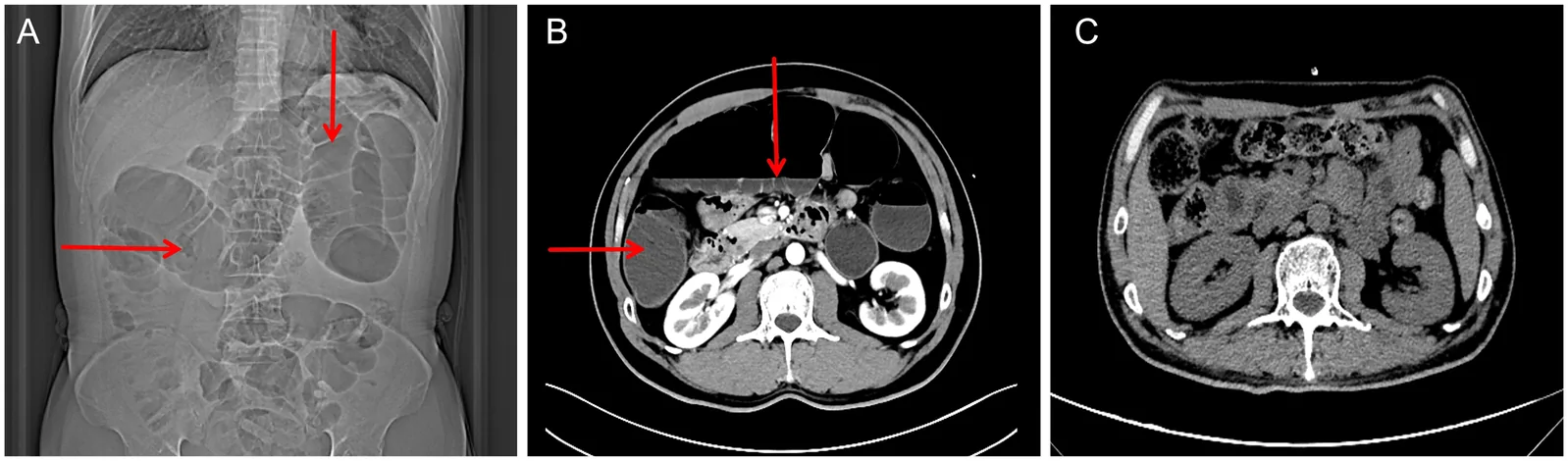
**(A)** (indicated by the arrow) shows dilated bowel loops on an abdominal X-ray. **(B)** (indicated by the arrow) shows diffusely dilated bowel loops with air-fluid levels on an axial contrast-enhanced abdominal CT. **(C)** Shows a follow-up axial abdominal CT obtained after chemotherapy and the subsequent relief of the patient’s bowel obstruction symptoms, demonstrating a marked reduction or disappearance of the dilated bowel loops and air-fluid levels compared to the pre-chemotherapy findings.

A 49-year-old man had undergone thymectomy for a thymoma discovered incidentally during a routine health examination three years previously (**Figure 1A**). Postoperative pathology revealed mixed-type thymoma (approximately 70% type B2 and 30% type B3; pT1aN0Mx; Masaoka stage IIB) (**Figure 2**). The patient declined adjuvant therapy. Follow-up examinations were unremarkable until six months before the current admission, when he was readmitted with progressive abdominal distension, vomiting, and constipation for two months, accompanied by dysgeusia for two weeks and diplopia that was milder in the morning and worse in the evening for five days. Contrast-enhanced chest CT demonstrated marked left pleural thickening (**Figure 1B**), and pleural biopsy confirmed metastatic thymoma. On hospital day 3, the patient’s symptoms worsened and progressed to intestinal obstruction. Abdominal radiography and contrast-enhanced abdominal CT showed diffuse bowel dilation with multiple air-fluid levels (**Figures 3A, B**). After mechanical obstruction was excluded by enema, colonoscopy, and diagnostic laparoscopy, acute intestinal pseudo-obstruction (AIPO) was considered. On hospital day 6, his condition further deteriorated, with new-onset blurred vision, visual field dimming, complete ophthalmoplegia, and complete loss of sweet and salty taste perception. The pyridostigmine test was positive. Brain MRI showed no significant abnormalities. Given the history of metastatic thymoma and multisystem involvement, PNS associated with thymoma recurrence was diagnosed. Given the rapid progression of multisystem PNS and the presumed autoimmune pathogenesis, intravenous methylprednisolone pulse therapy was initiated as an immunosuppressive intervention. No response was observed after two consecutive days of methylprednisolone pulse therapy (500 mg/day), and further pulses were refused by the family because of adverse reactions. Subsequently, the patient received CAP chemotherapy (doxorubicin 90 mg, cyclophosphamide 900 mg, and cisplatin 90 mg; every 21 days for five cycles), in combination with symptomatic pyridostigmine bromide (60 mg orally three times daily). The patient voluntarily stopped pyridostigmine after the ocular symptoms resolved. Clinical improvement occurred sequentially. After the first chemotherapy cycle, diplopia and blurred vision improved. By the end of the second cycle, ocular symptoms resolved completely, eye movements returned to normal, taste began to recover with partial restoration of salty taste, and vomiting markedly decreased. By the end of the third cycle, taste function was largely restored. Between the fourth and fifth cycles, the patient regained spontaneous flatus and defecation while recuperating at home. After completing five cycles, follow-up CT showed a significant reduction in left pleural thickening (**Figure 1C**) and near-complete resolution of bowel dilation (**Figure 3C**). Clinically, mental status, ocular symptoms, taste and AIPO symptoms recovered fully. Autoantibody testing by cell-based assay after completion of five treatment cycles showed positivity for acetylcholine receptor antibody (AChR-Ab; titer 1:10), anti-MURC antibody (titer 1:10), and anti-recoverin antibody (titer 1:10). Testing for ganglionic acetylcholine receptor antibody (gAChR-Ab), anti-titin, anti-RyR, anti-MuSK, and a paraneoplastic antibody panel (including Ma, Hu, Ri, and CV2) was negative.

**Figure 4:**
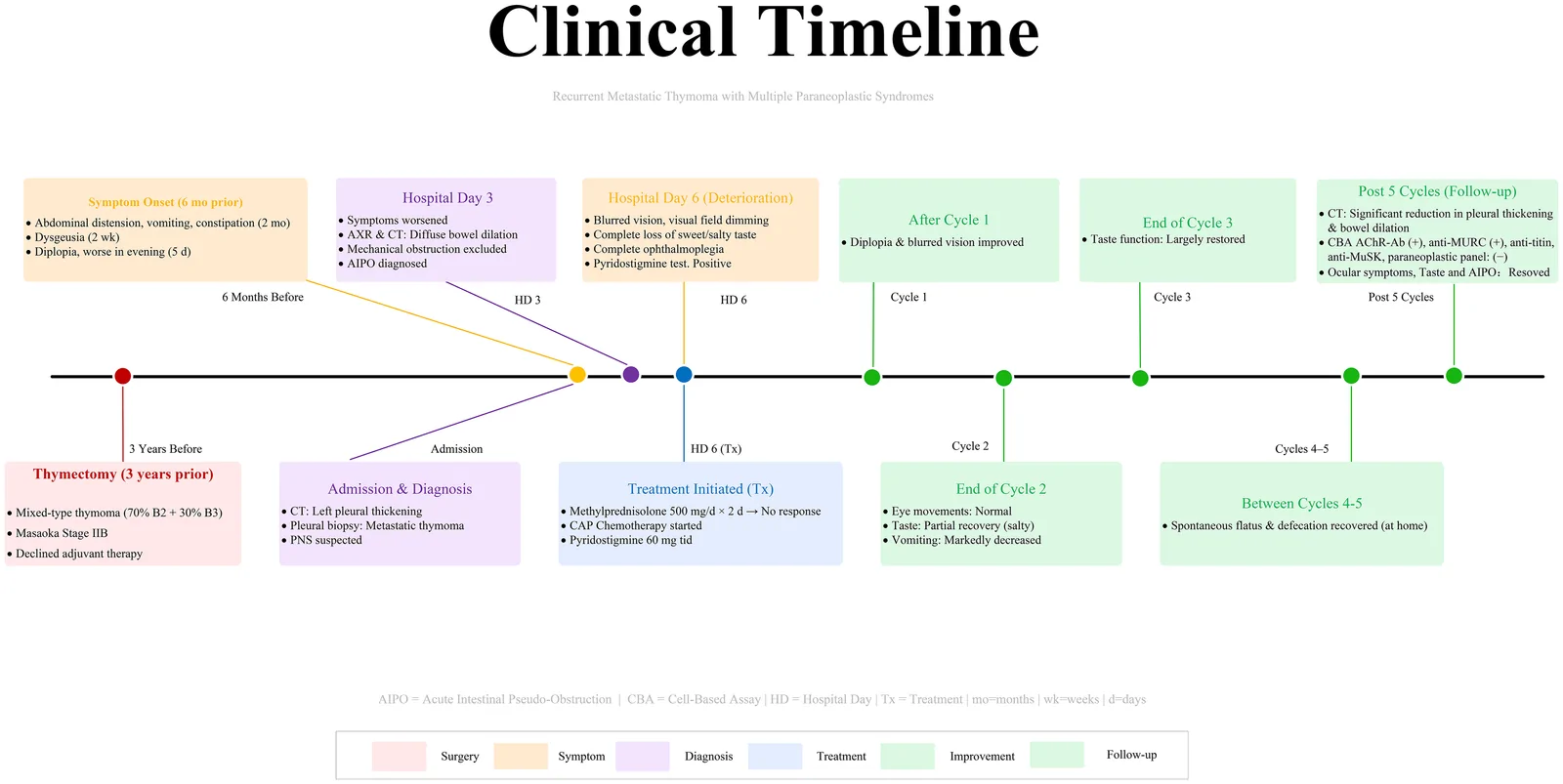
Timeline of clinical course. This timeline illustrates the patient’s entire diagnostic and therapeutic course, spanning from thymectomy for thymoma performed three years earlier, to re-admission six months ago due to multisystem symptoms, during which pleural metastasis of thymoma was confirmed, followed by the complex diagnostic workup and management of thymoma-associated paraneoplastic neurological syndromes (PNS), and ultimately leading to substantial symptomatic improvement.

Following completion of five cycles of CAP chemotherapy, the patient was discharged and continued regular outpatient follow-up. The current management plan includes periodic chest CT surveillance every 3 months for the first year to monitor for disease recurrence, with ongoing neurologic and gastrointestinal symptom assessment. Long-term oncologic follow-up is coordinated with the medical oncology team. The patient is in good condition, with no signs of disease progression. The intricate clinical trajectory of this case, encompassing the initial diagnosis, recurrence, and multi-system involvement, is best appreciated in a chronological timeline (**Figure 4**).

## Discussion

Thymoma-associated paraneoplastic syndromes (PNS) are commonly explained by a model centered on impaired autoimmune regulator (AIRE) function. This model proposes that AIRE deficiency permits the escape of autoreactive T cells from central tolerance, facilitates aberrant autoantigen presentation, and alters the tumor immune microenvironment (10). MG most commonly affects skeletal muscle through antibodies against the muscle-type AChR, whereas gastrointestinal involvement is uncommon, even though gastrointestinal smooth muscle expresses muscarinic AChRs (2, 3, 11). In the present case, ocular involvement, including diplopia and ophthalmoplegia, together with a positive pyridostigmine test and AChR antibody positivity, served as a diagnostic reference for MG. Unfortunately, due to financial and facility constraints, we were unable to perform serial antibody testing prior to and during the patient’s treatment, thereby forfeiting the opportunity for baseline comparison. Nevertheless, the clinical response itself served as the most compelling diagnostic evidence. Moreover, these antibody results were obtained following five cycles of CAP chemotherapy.

Paraneoplastic gastrointestinal dysmotility encompasses a broad clinical spectrum, ranging from Ogilvie’s syndrome (acute colonic pseudo-obstruction, ACPO) to AIPO (13). ACPO typically involves the colon predominantly and has been associated with autoimmune autonomic ganglionopathy mediated by ganglionic acetylcholine receptor antibodies (gAChR-Ab). However, the absence of gAChR-Ab does not rule out paraneoplastic gastrointestinal dysmotility. For instance, in Dhamija et al.’s series of 24 patients with autonomic gastrointestinal involvement, only about 11 were positive for ganglionic acetylcholine receptor antibodies. Similarly, Alnajjar et al. reported a case that was also negative for these antibodies (12). AIPO generally affects the colon, small bowel, or a more extensive portion of the digestive tract. The main mechanism involves antineuronal antibodies attacking the intrinsic enteric nervous system, namely the myenteric and submucosal plexuses along with interstitial cells of Cajal, which disrupts normal peristalsis. The extrinsic autonomic system can also be involved, further impairing gastrointestinal motility (5). In thymoma-associated PNS, several antibodies have been implicated in gastrointestinal dysmotility, including gAChR-Ab, N-type voltage-gated calcium channel (VGCC) antibodies, anti-CV2/CRMP5, anti-Hu, and muscle-type AChR-Ab (14, 15).

In the present case, imaging demonstrated diffuse small-bowel and colonic dilation (**Figures 3A, B**) rather than isolated colonic distension typical of classic Ogilvie’s syndrome. This pattern suggests that the autoimmune process affected multiple levels of the gut neuromuscular axis, consistent with the multisystem nature of the concurrent PNS. Notably, gAChR-Ab was undetectable even after clinical recovery, while seronegativity does not exclude prior autonomic ganglionitis or antibody levels below assay sensitivity, the concurrent muscle-type AChR-Ab positivity and negative paraneoplastic panel (Ma, Hu, Ri, CV2) indicate that alternative antibody-mediated mechanisms may contribute to enteric dysmotility in thymoma-associated PNS beyond the classic gAChR-Ab pathway. Although muscle-type AChR antibody positivity represents a possible contributing factor to enteric nervous system dysfunction, particularly given the structural homology between muscle-type and ganglionic AChRs (12). However, this remains speculative, as the precise pathogenic mechanism linking AChR-Ab to AIPO in the absence of detectable gAChR-Ab is currently unproven, and other immune-mediated pathways cannot be excluded. Importantly, the absence of a mechanical obstruction on diagnostic laparoscopy further supports a functional etiology and is consistent with thymoma-associated AIPO.

The diagnostic laparoscopy in this case highlights a recurring clinical dilemma. AIPO can initially mimic a surgical acute abdomen, and a subset of patients undergo exploratory surgery before pseudo-obstruction is recognized (5). This carries particular risk in patients with MG, in whom operative stress and anesthesia can precipitate deterioration and even myasthenic crisis. These considerations reinforce the importance of early multidisciplinary collaboration. Prompt integration of surgical assessment with neuroimmunological evaluation may help avoid unnecessary invasive procedures and reduce avoidable perioperative complications.

The patient’s dysgeusia and visual symptoms were also compatible with the antibody profile. Taste receptor cells depend on cholinergic signaling for normal transduction (16), and dysgeusia has been reported in association with AChR antibody positivity and may correlate with disease activity in some cases (7). In addition, anti-recoverin antibodies are classically linked to paraneoplastic retinopathy and may present with blurred vision and visual field disturbances (17). In the present case, although the patient reported blurred vision and visual field dimming, formal ophthalmologic evaluation including electroretinography and detailed retinal imaging was not performed due to resource limitations. Consequently, while anti-recoverin positivity was detected, its clinical significance in this patient remains uncertain. We are also unable to definitively attribute these visual complaints to anti-recoverin-mediated pathological changes. We can only speculate that the patient’s related symptoms may be associated with this antibody. However, in this patient, concurrent AChR-Ab and anti-recoverin positivity, together with the abrupt onset of dysgeusia and visual complaints, provided a serologic pattern consistent with multisystem PNS. Importantly, the presence of these antibodies does not establish direct pathogenicity; rather, they may serve as immunologic markers that correlate with the observed clinical phenotype. The temporal relationship between antibody detection and symptom onset suggests an association, though the exact causal contribution of each antibody to specific manifestations remains to be elucidated. Additionally, symptoms appeared before the initiation of chemotherapy (the patient had already presented with abdominal distension for 2 months, dysgeusia for 2 weeks, and diplopia for 5 days at admission), thus excluding CAP chemotherapy-induced acute gastrointestinal and visual toxicity. The overall clinical pattern, including symptom improvement after systemic therapy, further supports the diagnosis of multiple thymoma-associated PNS.

Compared with previously reported cases, the present case illustrates that multiple PNS manifestations may cluster within a relatively short time frame rather than developing gradually over years. Based on the two cases of multiple PNS reported by Anderson et al. and the case reported in this paper, common features can be identified: patients with multiple PNS symptoms tend to experience a concentrated outbreak within a short period of three months to half a year, rather than being spaced out over several years. In the cases reported by Anderson et al., the patient developed partial loss of taste, dysphagia, and blurred vision successively within about three months, followed shortly by severe abdominal distension, vomiting, AIPO, and other symptoms. In this paper, the patient’s related symptoms erupted successively within two months. In B2/B3 type thymoma, reduced AIRE expression and dysregulated antigen presentation may permit a rapid expansion of autoreactive clones, potentially producing near-simultaneous immune injury at several targets, including the neuromuscular junction, enteric plexus, and taste pathways (16). Particularly, in thymoma-associated PNS, AIPO and dysgeusia have been reported to precede classic MG manifestations in some patients (5, 6). A multi-institutional study by Kabasawa et al. on taste disorders in myasthenia gravis (MG) revealed that MG-related taste disorders occurred in approximately 2.4% of patients (9/371), all of whom were diagnosed with thymoma (8). Notably, in five patients, MG symptoms developed 2–3 months after the onset of taste disorders. This indicates that when dysgeusia and MG occur sequentially or simultaneously, they are largely closely related to thymoma. Even when a patient presents with early-onset dysgeusia, it should serve as a warning sign for thymoma. Coincidentally, the patient in this article with pleural metastatic thymoma also presented with gastrointestinal symptoms as the initial manifestation, followed by the development of dysgeusia. Therefore, new-onset dysgeusia or unexplained gastrointestinal pseudo-obstruction, especially when accompanied by ocular symptoms, should prompt consideration of thymoma new onset, recurrence or metastasis in the differential diagnosis.

A striking feature in this case was the sequential recovery pattern, with ocular symptoms improving first, followed by taste, while gastrointestinal function improved most slowly. This gradient may reflect differences in the reversibility and depth of immune-mediated injury across organ systems. MG often represents a functional transmission defect at the postsynaptic membrane that can recover with reduced immune activity and appropriate therapy. Taste dysfunction may improve relatively quickly because taste bud cells undergo rapid turnover and can regenerate once immune injury abates (18). In contrast, AIPO may involve autoimmune ganglionitis of the myenteric plexus, with potential irreversible loss or degeneration of enteric neurons (5). Gastrointestinal motility also depends on coordinated autonomic input, enteric neuronal integrity, and smooth muscle function (19). These considerations may explain why gastrointestinal function was the last to improve in this case.

## Limitations

Electrophysiological testing, quantitative taste evaluation, objective ophthalmologic testing, and standardized gastrointestinal motility studies could not be carried out because of financial and facility constraints. Ocular myasthenia gravis was clinically suspected based on the characteristic diurnal fluctuation of diplopia and extraocular muscle weakness, with worsening toward evening, together with a positive pyridostigmine trial, and was later supported by a post-treatment positive AChR-Ab result. AIPO was considered after imaging and laparoscopic findings excluded mechanical obstruction, in the setting of known metastatic thymoma with presumed paraneoplastic involvement. Baseline autoantibody assays were not available locally before treatment initiation. Finally, as this is a single case, the findings should be interpreted cautiously until corroborated by larger case series.

## Conclusion

This case describes a rare overlap of three thymoma-associated paraneoplastic syndromes: myasthenia gravis with diplopia and ophthalmoplegia, AIPO, and dysgeusia. These manifestations occurred in the setting of recurrent metastatic thymoma and were accompanied by multiple potentially related autoantibody positivities. After five cycles of CAP chemotherapy and short-term pyridostigmine therapy, radiographic evidence of disease decreased, and the associated neurologic, sensory, and gastrointestinal symptoms improved sequentially. This case indicates that multisystem PNS may debut and cluster during thymoma recurrence. Additionally, these symptoms showed sequential resolution following treatment. This case also suggests that in clinical practice, when encountering patients who present with unexplained gastrointestinal motility disorders (such as AIPO) or dysgeusia at an early stage, attention should be paid to the differential diagnosis of new-onset thymoma, or vigilance should be exercised regarding the possibility of postoperative recurrence or metastasis of thymoma. Early neuroimmunological evaluation and coordinated multidisciplinary care are essential to distinguish AIPO from surgical acute abdomen and to guide anti-tumor treatment for suspected enteric nervous system involvement.

## Data Availability

The original contributions presented in the study are included in the article/supplementary material. Further inquiries can be directed to the corresponding author.
